# Death from COVID-19 in a Fully Vaccinated Subject: A Complete Autopsy Report

**DOI:** 10.3390/vaccines11010142

**Published:** 2023-01-09

**Authors:** Massimiliano Esposito, Giuseppe Cocimano, Fabrizio Vanaria, Francesco Sessa, Monica Salerno

**Affiliations:** 1 Department of Medical, Surgical and Advanced Technologies “G.F. Ingrassia”, University of Catania, 95121 Catania, Italy; 2 Department of Mental and Physical Health and Preventive Medicine, University of Campania “Vanvitelli”, 80121 Napoli, Italy; 3 State Police, Health Service Department, Ministry of Interior, 95121 Catania, Italy

**Keywords:** COVID-19, forensic autopsy, vaccine efficacy, public health

## Abstract

A correctly implemented and widely accepted vaccination campaign was the only truly effective weapon to reduce mortality and hospitalizations related to COVID-19. However, it was not 100% effective and has not eliminated COVID-19. Even though more than 60% of the worldwide population is fully vaccinated (meaning that these subjects have completed the recommended vaccine cycle), subjects continue to die from COVID-19, particularly in the presence of comorbidities. In this scenario, autopsies play a crucial role in understanding the pathophysiological mechanisms of SARS-CoV-2 in vaccinated subjects and adapting therapies accordingly. This case report analyzes the death of a fully vaccinated patient who suffered from comorbidities and died from COVID-19; we provide a complete autopsy data set. On microscopic examination, the lungs showed massive interstitial pneumonia, areas of inflammation with interstitial lympho-plasma cell infiltrate, and interstitial edema. The liver showed granulocytes within the hepatic parenchyma. All these elements were consistent with previous published data on unvaccinated patients who had died from COVID-19. The present study is the first that analyzes, through a complete autopsy and a microscopic analysis of all organs, a death related to COVID-19 despite vaccine administration. In this regard, to the best of our knowledge, no other studies have been published reporting a complete autopsy. This study reports, on the one hand, the importance of vaccination programs in the fight against COVID-19, and, on the other hand, it hypothesizes that the vaccine does not offer complete immunity to SARS-CoV-2, particularly in elderly subjects with comorbidities.

## 1. Introduction

The increase in variants of SARS-CoV-2 leads to an increase in the infectivity of the virus and a reduction in vaccine efficacy, both due to viral immuno-resistance and the physiological reduction of the antibody level against the virus. The increase in variants raises concerns and alarmism in international health systems because of their greater transmissibility [1] and their greater “immune evasion”. In fact, the increase in infections and hospitalizations of vaccinated individuals probably derives from a combination of a decrease in vaccination efficacy over time, and a reduction in vaccination efficiency against new variants [2].

COVID-19 vaccine efficiency estimates range from 55 to 70% after the first dose, with little variation by vaccine or age group [3]. At first, the double dose of the COVID-19 vaccine had an efficacy varying between approximately 65% and 95%, producing a reduction in hospitalization of 75–85% and a reduction in mortality of 95–99%. A 35–50% reduction in transmission and risk of SARS-CoV-2 positivity was also found [4,5]. According to some authors, 14/20 days after the first dose, the effectiveness of the COVID-19 vaccine was about 46%, with a reduction in symptomatic disease of about 57%, hospitalizations were reduced by approximately 74%, severe disease by around 62%, and mortality by 72% [6]. On the other hand, 7 days after the second dose, the efficacy of the COVID-19 vaccine for documented infections was around 92%, 94% for symptomatic disease, 87% for the reduction in hospitalization, and 92% for severe disease [7]. In Chile, out of a cohort of 10.2 million people, the effectiveness of the vaccine was 66%, with a 90% reduction in hospitalization, and an 86.3% reduction in mortality [8]. However, due to the increase in COVID-19 variants, such as the omicron variant (B.1.1.529), the efficacy of the vaccine has recently changed dramatically. A case–control study [9] showed that the vaccine efficacy after two doses of BNT162b2 or ChAdOx1 was 65%, and 74%, respectively, decreasing with time. Primary immunization with a double dose of vaccine (ChAdOx1 nCoV-19 or BNT162b2) provided limited protection against symptomatic disease caused by the omicron variant. A BNT162b2 or mRNA-1273 booster after the primary course of ChAdOx1 nCoV-19 or BNT162b2 substantially increased protection, but it decreased proportionally over time. According to Hammerman et al. [10], the efficacy of the vaccine against COVID-19 was estimated at 82% among patients aged between 16 and 64 years, and at 60% in the over 65s. Furthermore, in patients who recovered from COVID-19 after hospitalization, the administration of at least one dose of the BNT162b2 vaccine was associated with a decrease in the risk of new COVID-19 infections.

Although it is known that the COVID-19 vaccine does not prevent death or the onset of serious disease, there are few forensic case reports concerning COVID-19 deaths despite the administration of the vaccine. It is important to note that the subject of our case had comorbidities that contributed to the death of the patient. This case report accurately describes the results of a complete autopsy in a fully vaccinated man, who had completed the vaccine cycle, who had died from COVID-19 in the presence of comorbidities [11]. This paper could be of great importance for the scientific community, to clarify how the COVID-19 vaccine does not mean total immunity from this disease, but a reduction in the possibility of contracting a serious disease or preventing death, strengthening the concept that the only weapon against COVID-19 is the mass vaccination of the entire population [12]. Vaccination does not necessarily mean total immunity particularly in elderly subjects with comorbidities, it means increased protection and an increased success rate to eradicate the SARS-CoV-2 infection. The efficacy of the vaccine also varies according to type from 70.4% for the ChAdOx1 nCoV-19 vaccine to 96% for the mRNA-1273 vaccine [13]. This case report wants to reiterate this concept to the scientific community and that, despite a complete vaccination, there will always be deaths. Therefore, it is essential to encourage vaccination campaigns and promote herd immunity.

## 2. Materials and Methods

### 2.1. Case Description

An 83-year-old man who suffered from heart failure, valvular heart disease, chronic obstructive pulmonary disease (COPD), diabetes mellitus, and chronic renal failure, was admitted to hospital with worsening dyspnea. The patient was fully vaccinated (he had received a double dose of the BNT162b2 (Comirnaty) mRNA vaccine, Pfizer, New York, NY, USA). He was admitted to the Emergency Department for an exacerbation of chronic heart failure, which was treated according to the guidelines of the European Society of Cardiology [14]. A nasopharyngeal swab tested negative for SARS-CoV-2 on admission to the hospital. After 11 days of hospitalization, the patient complained of worsening dyspnea, the health workers again took a molecular swab that gave a positive result for COVID-19 [15,16]. Since the first PCR swab obtained at the time of hospitalization was negative and the patient had no flu symptoms or symptoms related to SARS-CoV-2, and the second swab, performed after 11 days of hospitalization, positive for COVID-19, the acquisition of COVID-19 was nosocomial and met the definition of hospital acquired infections [17]. Although the patient was treated for COVID-19 according to the guidelines, he died after 18 days. No ethical committee approval was required. Written informed consent was obtained from the deceased’s relatives.

### 2.2. Laboratory Investigation

On admission to hospital, a serological test for antibodies to SARS-CoV-2 was negative for IgM, while the presence of IgG antibodies was demonstrated (25.40 AU/mL; reference values < 0.8–1.2 U/mL; Roche ECLIATM). On the second day of hospitalization, a further serological examination was performed, which gave a negative result while it demonstrated the stability of the value of IgG antibodies (25.40 AU/mL; reference values < 0.8–1.2 U/mL; Roche ECLIATM). After death, genotyping of the SARS-CoV-2 variant was carried out that confirmed the presence of the delta variant (B.1.617.2); this was consistent with the spread of the virus in Italy in that period.

### 2.3. Autopsy Findings

**Figure 1:**
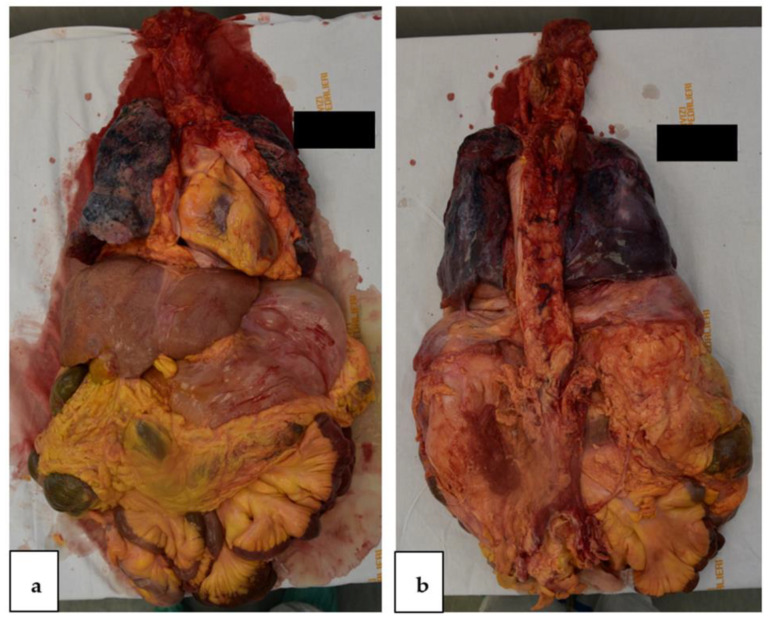
Letulle block, anterior (**a**), posterior (**b**).

The autopsy was conducted according to the recommendations provided for COVID-19 autopsies and for those with a high biological risk [18]. The autopsy was conducted according to the Letulle technique, thus reducing environmental contamination (Figure 1). This technique consisted of carrying out an en bloc resection of all the cervical, thoracic, and abdominal organs to prevent the aerosolization of potentially contaminated biological fluids [19,20]. The coronaries were sectioned and all showed regular lumen and course, slight wall thickening, and no stenoses. 

### 2.4. Histological Analysis

**Figure 2:**
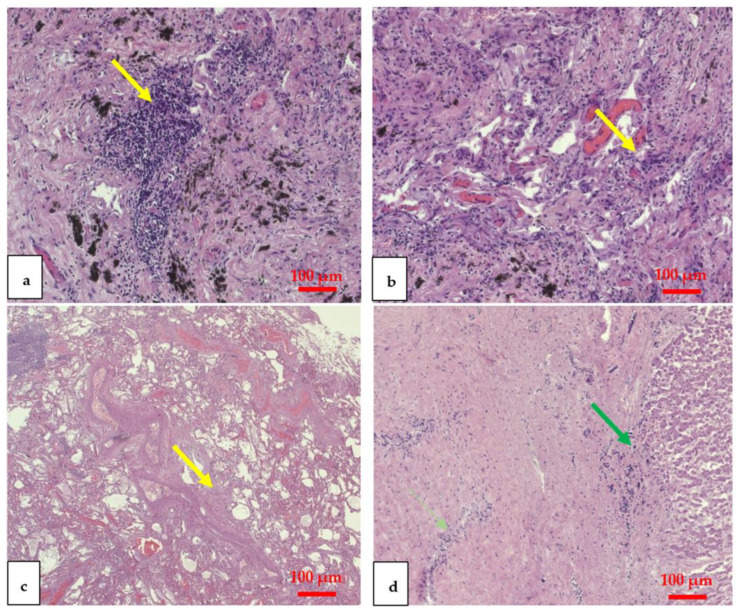
H&E x 10. Lungs showed interstitial lympho-plasma cell infiltrate, interstitial edema, and interstitial fibrosis ((**a**–**c**), yellow arrows). The liver showed granulocytes within the parenchyma ((**d**), green arrow).

All the organs were fixed in 10% buffered formalin; after washing, each organ was sampled, and the sections were embedded in paraffin. Using a microtome (Dako, Glostrup, Denmark) paraffin-embedded samples were cut into thin slices of 5 micrometers that were stained with hematoxylin and eosin (H&E). The slides were viewed under a Zeiss Axioplan light microscope and photographed with a Zeiss AxioCam MRc5 digital camera (Carl Zeiss, Oberkochen, Germany). At H&E staining, the lungs showed areas of chronic emphysema with massive interstitial pneumonia. The lungs showed numerous areas of inflammation with interstitial lympho-plasma cell infiltrate, and an interstitial edema. An increase in pulmonary interstitial fibrosis was also found. The H&E-stained liver showed areas of granulocytes within the liver parenchyma (Figure 2).

Table 1 summarizes the macroscopic and microscopic characteristics of the case report.

### 2.5. Immunohistochemical Analysis

**Figure 3:**
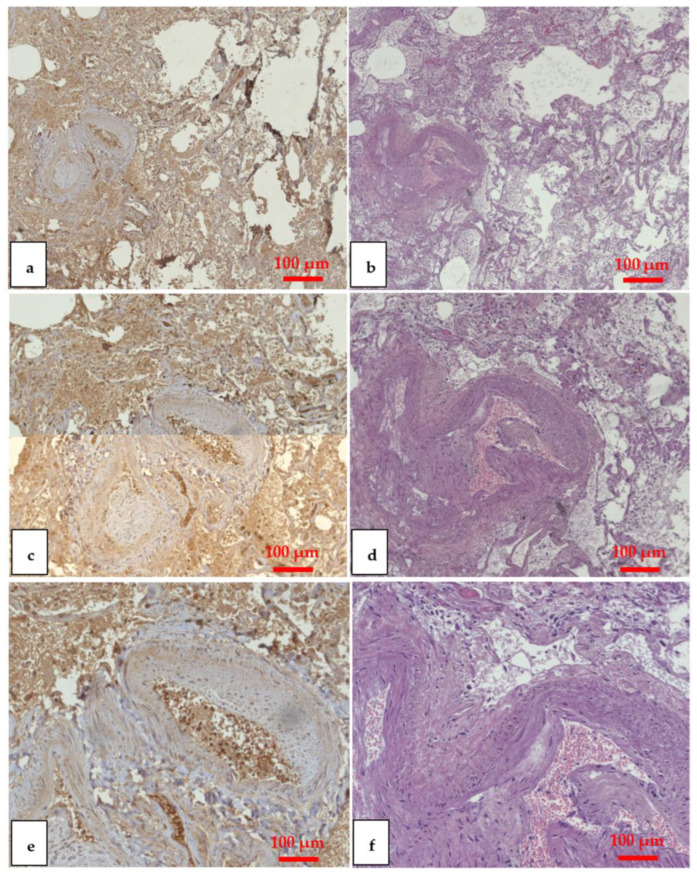
Immunohistochemical (IHC) and H&E lower lobe of the left lung. The sample shows a strong positivity (+++) to the anti-COVID nucleocapsid antibody, a clear sign of death from COVID-19, at IHC and H&E x5 (**a**,**b**), x10 (**c**,**d**), x20 (**e**,**f**).

The formalin-fixed paraffin-embedded (FFPE) tissues were cut into 2~3 μm sections and collected on poly-L-lysine-coated glass slides. The paraffin sections were mounted on slides covered with 3-aminopropyltriethoxysilane (Fluka, Buchs, Switzerland). Pre-treatment was necessary to facilitate antigen retrieval and to increase membrane permeability to antibodies. The primary antibody was applied in a 1:500 ratio for all antibodies and incubated for 120 min at 20 °C. The detection system used was the LSAB+ kit (Dako, Copenhagen, Denmark), a refined avidin–biotin technique in which a biotinylated secondary antibody reacts with several peroxidase-conjugated streptavidin molecules. The sections were counterstained with Mayer’s hematoxylin, dehydrated, cover slipped and observed under a Leica DM4000B optical microscope (Leica, Cambridge, UK). For the immunohistochemical investigation, the anti-COVID nucleocapsid antibody (anti-Coronavirus - FIPV3-70, Santa Cruz Biotechnology, Inc., Dallas, TX, USA), specific for SARS-CoV-2 was used (Figure 3) [21]. 

A quality score was assigned depending on positivity. The ranks ranged from "negative" (mostly marked as "-") to "positive", which can be denoted by a different number of "+" depending on how many other categories are found between these parameters. Therefore, depending on the difference in positivity, this classification was remodeled into "negative" (-), "very weak" (+/-), "weak" (+), "moderate" (++), "strong" ( +++), and “very strong” (++++) [22,23,24,25].

## 3. Discussion

A vaccination campaign represents the only truly effective method for reducing COVID-19-related mortality, even though COVID-19 has not been eliminated. In the present case, although the subject was fully vaccinated, he died from COVID-19 in the presence of numerous comorbidities. A recent study has, in fact, analyzed about 5 million subjects with double-vaccination doses. Of these subjects, 2031 had died from COVID-19 despite the double dose, and of these, 81 deaths occurred within 14 days of vaccination. Several risk factors were established, such as increasing age, male sex, cancer, neurological disorders, and kidney disease [26]. Cancer is also a COVID-19-related risk factor for death despite vaccination. In fact, Heudel et al. [27] analyzed this mortality in more than 2000 cancer patients who had received at least one dose of the vaccine (one to three). Thirty-nine of the patients contracted SARS-CoV-2 with a median of 27 days after vaccination, and of these, six died; risk factors were age and metastatic disease.

Autopsies play a crucial role in understanding the pathophysiological mechanisms of COVID-19, with the aim of adapting future therapies accordingly. Autopsies conducted on subjects who died of COVID-19 showed pulmonary embolism, diffuse alveolar damage (DAD), hyaline membranes, thromboembolism, and interstitial edema with heterogeneous inflammatory tissue. Centrilobular necrosis and moderate portal or lobular inflammation are described in the liver [28].

In deaths due to the COVID-19 vaccine, it is necessary to report myocarditis, and again vaccine-induced immune thrombotic thrombocytopenia syndrome (VITT/ VIPIT), with rosary-like systemic thrombosis, especially of the encephalic and gastro-intestinal systems [29,30,31,32].

This case report, in fact, analyzes the death of a patient who suffered from comorbidities and died from COVID-19 despite a double vaccination dose. The patient was hospitalized for an exacerbation of heart failure: at hospitalization the nasopharyngeal swab tested negative for SARS-CoV-2 infection, while the serological test demonstrated the presence of IgG with negative data for IgM presence. However, it is necessary to specify that while the positivity to IgG confirmed the vaccine, the negativity to the PCR and IgM tests did not exclude with certainty the presence of an infection already in progress. Some authors have confirmed that in the early stages of SARS-CoV-2 contraction, there is a 5-day “window” in which the PCR or antibody test is negative [33]. After 11 days of hospitalization, the patient contracted SARS-CoV-2 and died after another 18 days of COVID-19. The patient suffered from multiple comorbidities: heart disease, COPD, diabetes mellitus, and kidney failure. During the autopsy, Letulle’s block was performed with subsequent organ fixation in formalin. Consistent with the studies of subjects who died from COVID-19 [28,34], on H&E staining, the lungs showed massive interstitial pneumonia, areas of inflammation with interstitial lympho-plasma cell infiltrate, and an interstitial edema. The liver showed granulocytes within the liver parenchyma.

A similar case report was published by Hansen et al. [35] and concerned an 86-year-old man with multiple comorbidities who died of COVID-19 4 weeks after vaccination. The autopsy showed acute bilateral bronchopneumonia, including bacterial overinflection: the histological examination showed no clear signs of COVID-19-related pneumonia, such as interstitial pneumonia and diffuse alveolar damage. However, unlike what Hansen et al. found [35], this case report is characterized by all the autopsy and histological elements of a COVID-19-related death.

Nevertheless, in general, the reduced efficacy of the COVID-19 vaccine in the general population has now been established, due to the combination of various factors including the increase in variants, the reduction of the vaccination campaign, and the loss of efficacy of the vaccine over time [36,37,38]. Moreover, the presence of comorbidities in elderly subjects could influence vaccine efficacy [39,40]. The increase in SARS-CoV-2 variants is also referred to as "immune evasion" and could increasingly lead to a decrease in the effectiveness of anti-viral strategies, as well as that of the vaccine [41,42,43]. In New York State, USA, out of more than 8,500,000 adults given the vaccine, nearly 151,000 cases of COVID-19 were recorded with approximately 14,500 hospitalizations, and the median efficacy of the vaccine was 96.9%. Subsequently, a progressive decrease in the efficacy of the vaccine was recorded from 93.4% to 74.2%; however, the efficacy against hospitalization remained high [41]. Individual factors can also change the efficacy of the vaccine, for example a different body mass index (BMI) could lead to a different vaccine response; obese subjects, in fact, have a decrease in vaccination efficacy [42]. Obviously, increasing age and comorbidities also decrease the effectiveness of the vaccine for the transmission of SARS-CoV-2, increasing the risks of hospitalization and mortality [43]. Andrews et al. [44], on the other hand, state that, by administering booster doses of the vaccines, the absolute effectiveness of the vaccine in preventing death or hospitalization varied from about 97 to 99%, in all age groups for at least 10 weeks. The authors concluded that in subjects over 50 years of age, a booster dose of the COVID-19 vaccine is still important in preventing hospitalization, serious illness, or death. These results appear to be shared by other studies as well [45,46]. Other authors affirm that administering boosters every 4–6 months to keep antibodies against COVID-19 high is currently not a winning strategy [47]. In fact, some studies clarify that by carrying out continuous boosters, an increase in the effectiveness of the vaccine is not recorded. Furthermore, the vaccination campaign would gather less support in the general population, this could also be due to the fear of side effects [48,49,50,51].

A recent study [52] highlighted that of 8084 patients with COVID-19 infections, 3% (245) died during the 4-month follow-up (January to April 2021), despite complete vaccination. Of these 245 patients, 191 deaths were COVID-19 related (COVID-19 deaths), furthermore, a vaccine difference was not recorded. The deceased patients were older (mean age 82 years vs. 57 years), and had severe comorbidities. Our study is consistent with this article and shows how people can die from COVID-19 despite a full vaccination.

As suggested, a broader epitope coverage is needed for the next generation of COVID-19 vaccines, providing protection against more variants. Moreover, it is necessary to exploit multiple vaccination platforms, thus the capacity of a specific vaccine, or combination, is exploited for a specific target of the population such as certain age groups, pregnant women, and immunocompromised subjects [53].

Furthermore, physicians must always promote vaccination campaigns, through appropriate information made to patients, and through timely support and encouragement [54].

The present study is the first that analyzes, through a complete autopsy and microscopic analysis of all organs, a COVID-19-related death even though the subject was fully vaccinated. It is important to remark that the subject was elderly and suffered from comorbidities that undoubtedly influenced his outcome. Other studies have been published but without a complete autopsy. This study also reaffirms the concept that vaccines do not offer complete immunity to SARS-CoV-2 and are not even 100% effective; therefore, it is necessary that the scientific community is always careful to keep this pandemic under control in light of the ongoing variants of SARS-CoV-2.

## 4. Conclusions

By carrying out a complete autopsy of a subject who died of COVID-19 despite a double vaccination dose, the present study reaffirms that an adequate vaccination campaign is essential in adults with many pathologies. In fact, as stated by Morens et al. [55], the latest events make it clear that SARS-CoV-2 is unlikely to be eliminated, let alone eradicated, which is why it will continue to circulate indefinitely in periodic epidemics and will become endemic. It is also necessary that research focuses on new vaccines that can be better adapted to a specific target population.

## Figures and Tables

**Table 1:** Main findings of all organs obtained during the autopsy.

Organ	Weight Grams (g) and Measurements Centimeters (cm)	Histological Findings
Brain	g 1470 cm 18 × 17 × 7	Perivasal edema and perineuronal edema
Heart	g 433 cm 12 × 9 × 5	Myofiber breakup and colliquative myocytolysis
Right lung	g 925 cm 23 × 11 × 9	Interstitial lympho-plasma cell infiltrate, interstitial edema, and interstitial fibrosis
Left lung	g 601 cm 22 × 10 × 7	
Liver	g 1085 cm 17 × 15 × 6	Intraparenchymal polymorphonuclear granulocytes
Spleen	g 95 cm 12 × 3 × 3	Stasis
Right kidney	g 45 cm 9 × 4 × 2	Large areas of connective tissue replacement of the parenchyma
Left kidney	g 35 cm 8 × 5 × 2	

## Data Availability

Not applicable.
